# Supplementary material for: Identifying Protein Features Responsible for Improved Drug Repurposing Accuracies Using the CANDO Platform: Implications for Drug Design
Source: Molecules. 2019 Jan 4;24(1):167. doi: 10.3390/molecules24010167 (PMC6337359; doi:10.3390/molecules24010167)
Supplement: Supplementary file 1 [file molecules-24-00167-s001.zip › fileS1.pdf]

MeSH heading	MeSH ID	Compound name	#compounds	approved	consensus score	consensus ratio
"Malaria, Cerebral"	MESH:D016779	zosuquidar	2	2	1.0	
"Malaria, Cerebral"	MESH:D016779	posaconazole	2	2	1.0	
"Malaria, Cerebral"	MESH:D016779	cinchoninecomma_monohydrochloridecomma_(9s)-isomer	2	2	1.0	
Malaria	MESH:D008288	cinchoninecomma_monohydrochloridecomma_(9s)-isomer	19	4	0.210526315789	
Malaria	MESH:D008288	quinine_ethylcarbonate	19	4	0.210526315789	
Malaria	MESH:D008288	isothipendyl	19	3	0.157894736842	
Malaria	MESH:D008288	posaconazole	19	3	0.157894736842	
Malaria	MESH:D008288	alcaftadine	19	3	0.157894736842	
Malaria	MESH:D008288	zosuquidar	19	3	0.157894736842	
Malaria	MESH:D008288	chlortetracycline	19	3	0.157894736842	
"Malaria, Falciparum"	MESH:D016778	zosuquidar	20	3	0.15	
"Malaria, Falciparum"	MESH:D016778	cinchoninecomma_monohydrochloridecomma_(9s)-isomer	20	3	0.15	
"Malaria, Falciparum"	MESH:D016778	posaconazole	20	3	0.15	
Malaria	MESH:D008288	furazabol	19	2	0.105263157895	
Malaria	MESH:D008288	methacycline	19	2	0.105263157895	
Malaria	MESH:D008288	clomocycline	19	2	0.105263157895	
Malaria	MESH:D008288	dimethylaminoethyl_reserpilinate_dihydrochloride	19	2	0.105263157895	
Malaria	MESH:D008288	danazol	19	2	0.105263157895	
Malaria	MESH:D008288	emedastine	19	2	0.105263157895	
Malaria	MESH:D008288	metacycline	19	2	0.105263157895	
Malaria	MESH:D008288	dapiprazole	19	2	0.105263157895	
Malaria	MESH:D008288	tigecycline	19	2	0.105263157895	
Malaria	MESH:D008288	demeclocycline	19	2	0.105263157895	
Malaria	MESH:D008288	clemizole_hydrochloride	19	2	0.105263157895	
"Malaria, Falciparum"	MESH:D016778	furazabol	20	2	0.1	
"Malaria, Falciparum"	MESH:D016778	sulfamethoxazole	20	2	0.1	
"Malaria, Falciparum"	MESH:D016778	pemetrexed	20	2	0.1	
"Malaria, Falciparum"	MESH:D016778	tetrahydrofolic_acid	20	2	0.1	
"Malaria, Falciparum"	MESH:D016778	dimethylaminoethyl_reserpilinate_dihydrochloride	20	2	0.1	
"Malaria, Falciparum"	MESH:D016778	danazol	20	2	0.1	
"Malaria, Falciparum"	MESH:D016778	sinophenin	20	2	0.1	
"Malaria, Falciparum"	MESH:D016778	isothipendyl	20	2	0.1	
"Malaria, Falciparum"	MESH:D016778	quinine_ethylcarbonate	20	2	0.1	
"Malaria, Falciparum"	MESH:D016778	sulfametomidine	20	2	0.1	
"Malaria, Falciparum"	MESH:D016778	alcaftadine	20	2	0.1	
"Malaria, Falciparum"	MESH:D016778	levomefolic	20	2	0.1	
"Malaria, Falciparum"	MESH:D016778	raltitrexed	20	2	0.1	
"Malaria, Falciparum"	MESH:D016778	clemizole_hydrochloride	20	2	0.1	
"Malaria, Falciparum"	MESH:D016778	pemetrexed_disodium	20	2	0.1	
Extensively Drug-Resistant Tuberculosis	MESH:D054908	phenolphthalein	3	2	0.666666666667	
"Tuberculosis, Meningeal"	MESH:D014390	nicogamol	4	2	0.5	
"Tuberculosis, Meningeal"	MESH:D014390	pyrazinoic_acid	4	2	0.5	
"Tuberculosis, Meningeal"	MESH:D014390	nicotinic_acid	4	2	0.5	
"Tuberculosis, Meningeal"	MESH:D014390	methaniazide	4	2	0.5	
"Tuberculosis, Meningeal"	MESH:D014390	dacarbazine	4	2	0.5	
"Tuberculosis, Lymph Node"	MESH:D014388	nicogamol	5	2	0.4	
"Tuberculosis, Lymph Node"	MESH:D014388	pyrazinoic_acid	5	2	0.4	
"Tuberculosis, Lymph Node"	MESH:D014388	nicotinic_acid	5	2	0.4	
"Tuberculosis, Lymph Node"	MESH:D014388	methaniazide	5	2	0.4	
"Tuberculosis, Lymph Node"	MESH:D014388	dacarbazine	5	2	0.4	
"Tuberculosis, Central Nervous System"	MESH:D020306	nicogamol	5	2	0.4	
"Tuberculosis, Central Nervous System"	MESH:D020306	pyrazinoic_acid	5	2	0.4	
"Tuberculosis, Central Nervous System"	MESH:D020306	nicotinic_acid	5	2	0.4	
"Tuberculosis, Central Nervous System"	MESH:D020306	methaniazide	5	2	0.4	
"Tuberculosis, Central Nervous System"	MESH:D020306	dacarbazine	5	2	0.4	
"Tuberculosis, Spinal"	MESH:D014399	nicogamol	6	2	0.333333333333	
"Tuberculosis, Spinal"	MESH:D014399	pyrazinoic_acid	6	2	0.333333333333	
"Tuberculosis, Spinal"	MESH:D014399	nicotinic_acid	6	2	0.333333333333	
"Tuberculosis, Spinal"	MESH:D014399	methaniazide	6	2	0.333333333333	
"Tuberculosis, Spinal"	MESH:D014399	dacarbazine	6	2	0.333333333333	
"Tuberculosis, Multidrug-Resistant"	MESH:D018088	phenolphthalein	13	3	0.230769230769	
"Tuberculosis, Pulmonary"	MESH:D014397	azithromycin	11	2	0.181818181818	
"Tuberculosis, Pulmonary"	MESH:D014397	erythromycylamine	11	2	0.181818181818	
"Tuberculosis, Pulmonary"	MESH:D014397	methacycline	11	2	0.181818181818	
"Tuberculosis, Pulmonary"	MESH:D014397	pyrazinoic_acid	11	2	0.181818181818	

"Tuberculosis, Pulmonary"	MESH:D014397	rifapentine	11	2	0.181818181818
"Tuberculosis, Pulmonary"	MESH:D014397	roxithromycin	11	2	0.181818181818
"Tuberculosis, Pulmonary"	MESH:D014397	dacarbazine	11	2	0.181818181818
"Tuberculosis, Pulmonary"	MESH:D014397	nicogamol	11	2	0.181818181818
"Tuberculosis, Pulmonary"	MESH:D014397	nicotinic_acid	11	2	0.181818181818
"Tuberculosis, Pulmonary"	MESH:D014397	methaniazide	11	2	0.181818181818
"Tuberculosis, Pulmonary"	MESH:D014397	rifampicin	11	2	0.181818181818
"Tuberculosis, Pulmonary"	MESH:D014397	chlortetracycline	11	2	0.181818181818
"Tuberculosis, Multidrug-Resistant"	MESH:D018088	diflunisal	13	2	0.153846153846
"Tuberculosis, Multidrug-Resistant"	MESH:D018088	balofloxacin	13	2	0.153846153846
"Tuberculosis, Multidrug-Resistant"	MESH:D018088	oxibendazole	13	2	0.153846153846
"Tuberculosis, Multidrug-Resistant"	MESH:D018088	nicotinic_acid	13	2	0.153846153846
"Tuberculosis, Multidrug-Resistant"	MESH:D018088	pyrazinoic_acid	13	2	0.153846153846
"Tuberculosis, Multidrug-Resistant"	MESH:D018088	lomefloxacin	13	2	0.153846153846
"Tuberculosis, Multidrug-Resistant"	MESH:D018088	besifloxacin	13	2	0.153846153846
"Tuberculosis, Multidrug-Resistant"	MESH:D018088	enrofloxacin	13	2	0.153846153846
"Tuberculosis, Multidrug-Resistant"	MESH:D018088	orbifloxacin	13	2	0.153846153846
"Tuberculosis, Multidrug-Resistant"	MESH:D018088	dacarbazine	13	2	0.153846153846
"Tuberculosis, Multidrug-Resistant"	MESH:D018088	nicogamol	13	2	0.153846153846
"Tuberculosis, Multidrug-Resistant"	MESH:D018088	methaniazide	13	2	0.153846153846
"Tuberculosis, Multidrug-Resistant"	MESH:D018088	sodium_thiosalicylate	13	2	0.153846153846
Tuberculosis	MESH:D014376	balofloxacin	18	2	0.111111111111
Tuberculosis	MESH:D014376	lomefloxacin	18	2	0.111111111111
Tuberculosis	MESH:D014376	cefalexin	18	2	0.111111111111
Tuberculosis	MESH:D014376	oxibendazole	18	2	0.111111111111
Tuberculosis	MESH:D014376	nicotinic_acid	18	2	0.111111111111
Tuberculosis	MESH:D014376	pyrazinoic_acid	18	2	0.111111111111
Tuberculosis	MESH:D014376	enrofloxacin	18	2	0.111111111111
Tuberculosis	MESH:D014376	besifloxacin	18	2	0.111111111111
Tuberculosis	MESH:D014376	lenampicillin	18	2	0.111111111111
Tuberculosis	MESH:D014376	orbifloxacin	18	2	0.111111111111
Tuberculosis	MESH:D014376	dacarbazine	18	2	0.111111111111
Tuberculosis	MESH:D014376	norfloxacin	18	2	0.111111111111
Tuberculosis	MESH:D014376	nicogamol	18	2	0.111111111111
Tuberculosis	MESH:D014376	methaniazide	18	2	0.111111111111
Tuberculosis	MESH:D014376	bacampicillin	18	2	0.111111111111
"Carcinoma, Large Cell"	MESH:D018287	guanadrel	6	2	0.333333333333
"Carcinoma, Large Cell"	MESH:D018287	4-hydroxycyclophosphamide	6	2	0.333333333333
"Carcinoma, Large Cell"	MESH:D018287	2-(2-diethylaminoethoxy)ethyl_2-ethyl-2-phenylbutyrate	6	2	0.333333333333
"Carcinoma, Large Cell"	MESH:D018287	sobuzoxane	6	2	0.333333333333
"Carcinoma, Large Cell"	MESH:D018287	exametazime	6	2	0.333333333333
"Carcinoma, Large Cell"	MESH:D018287	pentoxyverine	6	2	0.333333333333
"Carcinoma, Large Cell"	MESH:D018287	guanadrel_sulfate	6	2	0.333333333333
"Carcinoma, Large Cell"	MESH:D018287	cycloserine	6	2	0.333333333333
"Carcinoma, Large Cell"	MESH:D018287	fosfomycin	6	2	0.333333333333
