# Supplementary material for: Identifying Protein Features Responsible for Improved Drug Repurposing Accuracies Using the CANDO Platform: Implications for Drug Design
Source: Molecules. 2019 Jan 4;24(1):167. doi: 10.3390/molecules24010167 (PMC6337359; doi:10.3390/molecules24010167)
Supplement: Supplementary file 1 [file molecules-24-00167-s001.zip › fileS2.pdf]

MeSH heading	MeSH ID	Compound name	#compounds approved	consensus score	consensus ratio
"Malaria, Cerebral"	MESH:D016779	voriconazole	2	2	1.0
"Malaria, Cerebral"	MESH:D016779	cinchoninecomma_monohydrochloridecomma_(9s)-isomer	2	2	1.0
"Malaria, Vivax"	MESH:D016780	quinacrine	3	2	0.666666666667
Malaria	MESH:D008288	voriconazole	19	4	0.210526315789
Malaria	MESH:D008288	cinchoninecomma_monohydrochloridecomma_(9s)-isomer	19	3	0.157894736842
Malaria	MESH:D008288	stanozolol	19	3	0.157894736842
Malaria	MESH:D008288	quinacrine	19	3	0.157894736842
Malaria	MESH:D008288	emedastine	19	3	0.157894736842
"Malaria, Falciparum"	MESH:D016778	sulfamethoxazole	20	3	0.15
"Malaria, Falciparum"	MESH:D016778	cinchoninecomma_monohydrochloridecomma_(9s)-isomer	20	3	0.15
"Malaria, Falciparum"	MESH:D016778	voriconazole	20	3	0.15
Malaria	MESH:D008288	furazabol	19	2	0.105263157895
Malaria	MESH:D008288	befunolol	19	2	0.105263157895
Malaria	MESH:D008288	minocycline	19	2	0.105263157895
Malaria	MESH:D008288	clemizole_hydrochloride	19	2	0.105263157895
Malaria	MESH:D008288	danazol	19	2	0.105263157895
Malaria	MESH:D008288	alprenolol	19	2	0.105263157895
Malaria	MESH:D008288	dapiprazole	19	2	0.105263157895
Malaria	MESH:D008288	perisoxalum	19	2	0.105263157895
Malaria	MESH:D008288	tigecycline	19	2	0.105263157895
Malaria	MESH:D008288	quinine_ethylcarbonate	19	2	0.105263157895
Malaria	MESH:D008288	sulfamethoxazole	19	2	0.105263157895
Malaria	MESH:D008288	demeclocycline	19	2	0.105263157895
Malaria	MESH:D008288	chlortetracycline	19	2	0.105263157895
"Malaria, Falciparum"	MESH:D016778	pemetrexed_disodium	20	2	0.1
"Malaria, Falciparum"	MESH:D016778	befunolol	20	2	0.1
"Malaria, Falciparum"	MESH:D016778	pemetrexed	20	2	0.1
"Malaria, Falciparum"	MESH:D016778	sulfisomidine	20	2	0.1
"Malaria, Falciparum"	MESH:D016778	danazol	20	2	0.1
"Malaria, Falciparum"	MESH:D016778	stanozolol	20	2	0.1
"Malaria, Falciparum"	MESH:D016778	sulfametomidine	20	2	0.1
"Malaria, Falciparum"	MESH:D016778	quinine_ethylcarbonate	20	2	0.1
"Malaria, Falciparum"	MESH:D016778	levomefolic	20	2	0.1
"Malaria, Falciparum"	MESH:D016778	raltitrexed	20	2	0.1
"Malaria, Falciparum"	MESH:D016778	furazabol	20	2	0.1
"Malaria, Falciparum"	MESH:D016778	tetrahydrofolic_acid	20	2	0.1
Extensively Drug-Resistant Tuberculosis	MESH:D054908	phenolphthalein	3	2	0.666666666667
Extensively Drug-Resistant Tuberculosis	MESH:D054908	furazolidone	3	2	0.666666666667
"Tuberculosis, Meningeal"	MESH:D014390	nicogamol	4	2	0.5
"Tuberculosis, Meningeal"	MESH:D014390	pyrazinoic_acid	4	2	0.5
"Tuberculosis, Meningeal"	MESH:D014390	nicotinic_acid	4	2	0.5
"Tuberculosis, Meningeal"	MESH:D014390	methaniazide	4	2	0.5
"Tuberculosis, Meningeal"	MESH:D014390	nicotinamide	4	2	0.5
"Tuberculosis, Lymph Node"	MESH:D014388	nicogamol	5	2	0.4
"Tuberculosis, Lymph Node"	MESH:D014388	pyrazinoic_acid	5	2	0.4
"Tuberculosis, Lymph Node"	MESH:D014388	nicotinic_acid	5	2	0.4
"Tuberculosis, Lymph Node"	MESH:D014388	methaniazide	5	2	0.4
"Tuberculosis, Lymph Node"	MESH:D014388	nicotinamide	5	2	0.4
"Tuberculosis, Central Nervous System"	MESH:D020306	nicogamol	5	2	0.4
"Tuberculosis, Central Nervous System"	MESH:D020306	pyrazinoic_acid	5	2	0.4
"Tuberculosis, Central Nervous System"	MESH:D020306	nicotinic_acid	5	2	0.4
"Tuberculosis, Central Nervous System"	MESH:D020306	methaniazide	5	2	0.4
"Tuberculosis, Central Nervous System"	MESH:D020306	nicotinamide	5	2	0.4
"Tuberculosis, Spinal"	MESH:D014399	nicogamol	6	2	0.333333333333
"Tuberculosis, Spinal"	MESH:D014399	pyrazinoic_acid	6	2	0.333333333333
"Tuberculosis, Spinal"	MESH:D014399	nicotinic_acid	6	2	0.333333333333
"Tuberculosis, Spinal"	MESH:D014399	methaniazide	6	2	0.333333333333
"Tuberculosis, Spinal"	MESH:D014399	nicotinamide	6	2	0.333333333333
"Tuberculosis, Pulmonary"	MESH:D014397	azithromycin	11	2	0.181818181818
"Tuberculosis, Pulmonary"	MESH:D014397	erythromycylamine	11	2	0.181818181818
"Tuberculosis, Pulmonary"	MESH:D014397	nicotinic_acid	11	2	0.181818181818
"Tuberculosis, Pulmonary"	MESH:D014397	pyrazinoic_acid	11	2	0.181818181818
"Tuberculosis, Pulmonary"	MESH:D014397	rifapentine	11	2	0.181818181818
"Tuberculosis, Pulmonary"	MESH:D014397	fluticasone_furoate	11	2	0.181818181818
"Tuberculosis, Pulmonary"	MESH:D014397	roxithromycin	11	2	0.181818181818

"Tuberculosis, Pulmonary"	MESH:D014397	nicogamol	11	2	0.181818181818
"Tuberculosis, Pulmonary"	MESH:D014397	methaniazide	11	2	0.181818181818
"Tuberculosis, Pulmonary"	MESH:D014397	nicotinamide	11	2	0.181818181818
"Tuberculosis, Pulmonary"	MESH:D014397	rifampicin	11	2	0.181818181818
"Tuberculosis, Pulmonary"	MESH:D014397	lacidipine	11	2	0.181818181818
"Tuberculosis, Multidrug-Resistant"	MESH:D018088	isocarboxazid	13	2	0.153846153846
"Tuberculosis, Multidrug-Resistant"	MESH:D018088	lomefloxacin	13	2	0.153846153846
"Tuberculosis, Multidrug-Resistant"	MESH:D018088	balofloxacin	13	2	0.153846153846
"Tuberculosis, Multidrug-Resistant"	MESH:D018088	nicotinamide	13	2	0.153846153846
"Tuberculosis, Multidrug-Resistant"	MESH:D018088	pyrazinoic_acid	13	2	0.153846153846
"Tuberculosis, Multidrug-Resistant"	MESH:D018088	nicotinic_acid	13	2	0.153846153846
"Tuberculosis, Multidrug-Resistant"	MESH:D018088	methaniazide	13	2	0.153846153846
"Tuberculosis, Multidrug-Resistant"	MESH:D018088	enrofloxacin	13	2	0.153846153846
"Tuberculosis, Multidrug-Resistant"	MESH:D018088	orbifloxacin	13	2	0.153846153846
"Tuberculosis, Multidrug-Resistant"	MESH:D018088	phenolphthalein	13	2	0.153846153846
"Tuberculosis, Multidrug-Resistant"	MESH:D018088	nicogamol	13	2	0.153846153846
"Tuberculosis, Multidrug-Resistant"	MESH:D018088	furazolidone	13	2	0.153846153846
"Tuberculosis, Multidrug-Resistant"	MESH:D018088	moxifloxacin	13	2	0.153846153846
"Tuberculosis, Multidrug-Resistant"	MESH:D018088	salcolex	13	2	0.153846153846
Tuberculosis	MESH:D014376	isocarboxazid	18	2	0.111111111111
Tuberculosis	MESH:D014376	lomefloxacin	18	2	0.111111111111
Tuberculosis	MESH:D014376	balofloxacin	18	2	0.111111111111
Tuberculosis	MESH:D014376	nicotinic_acid	18	2	0.111111111111
Tuberculosis	MESH:D014376	ranimustine	18	2	0.111111111111
Tuberculosis	MESH:D014376	methaniazide	18	2	0.111111111111
Tuberculosis	MESH:D014376	enrofloxacin	18	2	0.111111111111
Tuberculosis	MESH:D014376	norfloxacin	18	2	0.111111111111
Tuberculosis	MESH:D014376	pefloxacin	18	2	0.111111111111
Tuberculosis	MESH:D014376	nicogamol	18	2	0.111111111111
Tuberculosis	MESH:D014376	pyrazinoic_acid	18	2	0.111111111111
Tuberculosis	MESH:D014376	moxifloxacin	18	2	0.111111111111
Tuberculosis	MESH:D014376	nicotinamide	18	2	0.111111111111
"Carcinoma, Large Cell"	MESH:D018287	antimony_tartrate	6	2	0.333333333333
"Carcinoma, Large Cell"	MESH:D018287	n-hydroxymethylsuccinimide	6	2	0.333333333333
"Carcinoma, Large Cell"	MESH:D018287	imidazolidinyl_ureacomma_monosodium_salt	6	2	0.333333333333
"Carcinoma, Large Cell"	MESH:D018287	guanadrel	6	2	0.333333333333
"Carcinoma, Large Cell"	MESH:D018287	glymor	6	2	0.333333333333
"Carcinoma, Large Cell"	MESH:D018287	pyridrol	6	2	0.333333333333
"Carcinoma, Large Cell"	MESH:D018287	2primecomma2prime-difluorodeoxycytidine_5prime-triphosphate	6	2	0.333333333333
"Carcinoma, Large Cell"	MESH:D018287	4-hydroxycyclophosphamide	6	2	0.333333333333
"Carcinoma, Large Cell"	MESH:D018287	clofarabine	6	2	0.333333333333
"Carcinoma, Large Cell"	MESH:D018287	guanadrel_sulfate	6	2	0.333333333333
"Carcinoma, Large Cell"	MESH:D018287	fosfomycin	6	2	0.333333333333
