# Supplementary material for: Identifying Protein Features Responsible for Improved Drug Repurposing Accuracies Using the CANDO Platform: Implications for Drug Design
Source: Molecules. 2019 Jan 4;24(1):167. doi: 10.3390/molecules24010167 (PMC6337359; doi:10.3390/molecules24010167)
Supplement: Supplementary file 1 [file molecules-24-00167-s001.zip › fileS3.pdf]

Approved drug name	CANDO ID	MeSH heading	MeSH ID
1-(4-chlorophenyl)-5-isopropyl-biguanide_hydrochloride	1663	Malaria	MESH:D008288
1-(4-chlorophenyl)-5-isopropyl-biguanide_hydrochloride	1663	"Malaria, Falciparum"	MESH:D016778
amodiaquine	2259	"Malaria, Falciparum"	MESH:D016778
amodiaquine	2259	Malaria	MESH:D008288
artesunate	891	Malaria	MESH:D008288
artesunate	891	"Malaria, Falciparum"	MESH:D016778
astemizole	1938	Malaria	MESH:D008288
atovaquone	1619	Malaria	MESH:D008288
azlocillin	1837	"Malaria, Falciparum"	MESH:D016778
chlorprothixene	1092	"Malaria, Falciparum"	MESH:D016778
ciprofloxacin	2662	Malaria	MESH:D008288
clindamycin	293	"Malaria, Falciparum"	MESH:D016778
cloquinate	2471	"Malaria, Falciparum"	MESH:D016778
cloquinate	2471	Malaria	MESH:D008288
cloquinate	2471	"Malaria, Vivax"	MESH:D016780
clotrimazole	185	"Malaria, Falciparum"	MESH:D016778
dapsone	526	Malaria	MESH:D008288
dapsone	526	"Malaria, Falciparum"	MESH:D016778
doxycycline	1439	Malaria	MESH:D008288
folic_acid	1886	"Malaria, Falciparum"	MESH:D016778
halofantrinum	763	Malaria	MESH:D008288
halofantrinum	763	"Malaria, Falciparum"	MESH:D016778
hydrochloridecomma_thioridazin	2142	"Malaria, Falciparum"	MESH:D016778
hydroxychloroquine	2306	Malaria	MESH:D008288
leucovorin	1741	"Malaria, Falciparum"	MESH:D016778
lumefantrine	1211	Malaria	MESH:D008288
mefloquine	1591	"Malaria, Vivax"	MESH:D016780
mefloquine	1591	"Malaria, Falciparum"	MESH:D016778
mefloquine	1591	Malaria	MESH:D008288
mefloquine	1591	"Malaria, Cerebral"	MESH:D016779
primaquine	1525	"Malaria, Vivax"	MESH:D016780
primaquine	1525	Malaria	MESH:D008288
propafenone	2442	"Malaria, Falciparum"	MESH:D016778
pyrimethamine	1233	Malaria	MESH:D008288
pyrimethamine	1233	"Malaria, Falciparum"	MESH:D016778
quinidine	1754	"Malaria, Falciparum"	MESH:D016778
quinidine	1754	Malaria	MESH:D008288
quinine	1751	"Malaria, Cerebral"	MESH:D016779
quinine	1751	Malaria	MESH:D008288
quinine	1751	"Malaria, Falciparum"	MESH:D016778
sulfadoxine	1159	"Malaria, Falciparum"	MESH:D016778
sulfadoxine	1159	Malaria	MESH:D008288
sulfafurazole	1763	"Malaria, Falciparum"	MESH:D016778
tetracycline	159	Malaria	MESH:D008288
3-ethyl-thio-isonicotamide	438	Tuberculosis	MESH:D014376
3-ethyl-thio-isonicotamide	438	"Tuberculosis, Multidrug-Resistant"	MESH:D018088
acidscomma_aminosalicyli	1245	Tuberculosis	MESH:D014376
acidscomma_aminosalicyli	1245	"Tuberculosis, Multidrug-Resistant"	MESH:D018088
amikacin	2645	Tuberculosis	MESH:D014376
bekanamycin	2290	Tuberculosis	MESH:D014376
bekanamycin	2290	"Tuberculosis, Multidrug-Resistant"	MESH:D018088
capreomycin	2028	"Tuberculosis, Pulmonary"	MESH:D014397
capreomycin	2028	Tuberculosis	MESH:D014376
capreomycin	2028	"Tuberculosis, Multidrug-Resistant"	MESH:D018088
chenodiol	1750	Tuberculosis	MESH:D014376
ciprofloxacin	2662	Tuberculosis	MESH:D014376
ciprofloxacin	2662	"Tuberculosis, Spinal"	MESH:D014399
clarithromycin	825	"Tuberculosis, Pulmonary"	MESH:D014397
cycloserine	1706	Tuberculosis	MESH:D014376
cycloserine	1706	"Tuberculosis, Multidrug-Resistant"	MESH:D018088
cycloserine	1706	"Tuberculosis, Pulmonary"	MESH:D014397
ethambutol	386	Tuberculosis	MESH:D014376
ethambutol	386	"Tuberculosis, Central Nervous System"	MESH:D020306
ethambutol	386	"Tuberculosis, Lymph Node"	MESH:D014388
ethambutol	386	"Tuberculosis, Multidrug-Resistant"	MESH:D018088

ethambutol	386	"Tuberculosis, Pulmonary"	MESH:D014397
ethambutol	386	"Tuberculosis, Spinal"	MESH:D014399
gatifloxacin	1371	"Tuberculosis, Multidrug-Resistant"	MESH:D018088
linezolid	1521	Extensively Drug-Resistant Tuberculosis	MESH:D054908
ofloxacin	1704	"Tuberculosis, Pulmonary"	MESH:D014397
ofloxacin	1704	"Tuberculosis, Multidrug-Resistant"	MESH:D018088
ofloxacin	1704	"Tuberculosis, Lymph Node"	MESH:D014388
ofloxacin	1704	Tuberculosis	MESH:D014376
orion_brand_of_entacapone	1862	"Tuberculosis, Multidrug-Resistant"	MESH:D018088
orion_brand_of_entacapone	1862	Extensively Drug-Resistant Tuberculosis	MESH:D054908
pasiniazid	282	"Tuberculosis, Multidrug-Resistant"	MESH:D018088
pasiniazid	282	Tuberculosis	MESH:D014376
pasiniazid	282	"Tuberculosis, Pulmonary"	MESH:D014397
pasiniazid	282	"Tuberculosis, Spinal"	MESH:D014399
pasiniazid	282	"Tuberculosis, Meningeal"	MESH:D014390
pasiniazid	282	"Tuberculosis, Lymph Node"	MESH:D014388
pasiniazid	282	"Tuberculosis, Central Nervous System"	MESH:D020306
prednisolone	2615	Tuberculosis	MESH:D014376
pyrazinamidum	1284	"Tuberculosis, Spinal"	MESH:D014399
pyrazinamidum	1284	"Tuberculosis, Multidrug-Resistant"	MESH:D018088
pyrazinamidum	1284	"Tuberculosis, Pulmonary"	MESH:D014397
pyrazinamidum	1284	Tuberculosis	MESH:D014376
pyrazinamidum	1284	"Tuberculosis, Lymph Node"	MESH:D014388
pyrazinamidum	1284	"Tuberculosis, Central Nervous System"	MESH:D020306
pyrazinamidum	1284	"Tuberculosis, Meningeal"	MESH:D014390
pyridofylline	2020	Tuberculosis	MESH:D014376
pyridofylline	2020	"Tuberculosis, Pulmonary"	MESH:D014397
rifabutin	302	"Tuberculosis, Pulmonary"	MESH:D014397
rifampin	1888	"Tuberculosis, Pulmonary"	MESH:D014397
rifampin	1888	"Tuberculosis, Spinal"	MESH:D014399
rifampin	1888	"Tuberculosis, Multidrug-Resistant"	MESH:D018088
rifampin	1888	"Tuberculosis, Meningeal"	MESH:D014390
rifampin	1888	"Tuberculosis, Central Nervous System"	MESH:D020306
rifampin	1888	Tuberculosis	MESH:D014376
rifampin	1888	"Tuberculosis, Lymph Node"	MESH:D014388
streptomycin	1719	Tuberculosis	MESH:D014376
streptomycin	1719	"Tuberculosis, Pulmonary"	MESH:D014397
streptomycin	1719	"Tuberculosis, Meningeal"	MESH:D014390
streptomycin	1719	"Tuberculosis, Central Nervous System"	MESH:D020306
streptomycin	1719	"Tuberculosis, Spinal"	MESH:D014399
tasmar	2145	Extensively Drug-Resistant Tuberculosis	MESH:D054908
tasmar	2145	"Tuberculosis, Multidrug-Resistant"	MESH:D018088
tretinoin	1504	Tuberculosis	MESH:D014376
viomycin	2599	Tuberculosis	MESH:D014376
adenosine_triphosphate	1637	"Carcinoma, Large Cell"	MESH:D018287
cyclophosphamide	2001	"Carcinoma, Large Cell"	MESH:D018287
gemcitabine	1005	"Carcinoma, Large Cell"	MESH:D018287
ifosfamide	811	"Carcinoma, Large Cell"	MESH:D018287
paclitaxel	776	"Carcinoma, Large Cell"	MESH:D018287
vinorelbine	1075	"Carcinoma, Large Cell"	MESH:D018287
