# Supplementary figures and images for: Identifying Protein Features Responsible for Improved Drug Repurposing Accuracies Using the CANDO Platform: Implications for Drug Design
Source: Molecules. 2019 Jan 4;24(1):167. doi: 10.3390/molecules24010167 (PMC6337359; doi:10.3390/molecules24010167)

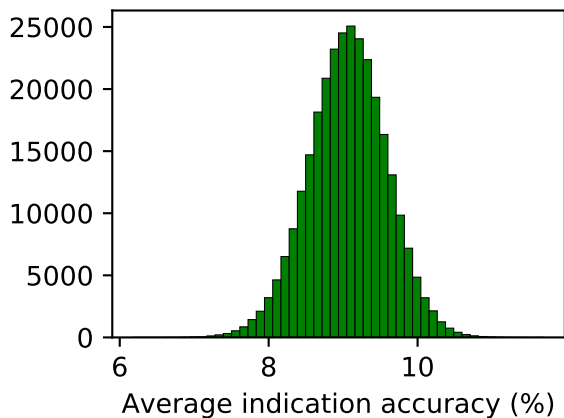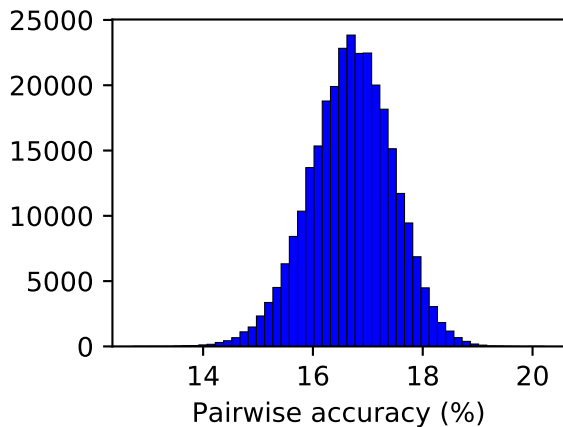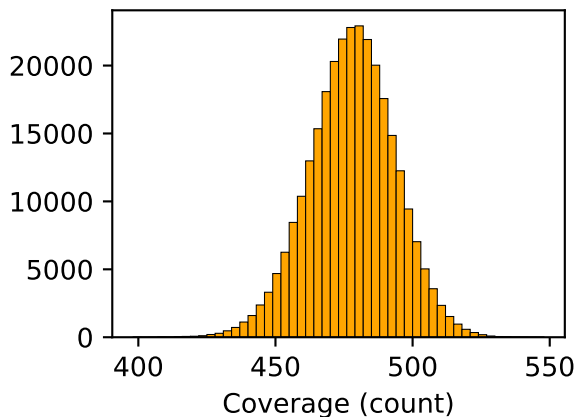

Supplement: Supplementary file 1 [file molecules-24-00167-s001.zip › figureS1.pdf]
